# Supplementary material for: Feasibility of a silicone vascular phantom replicating real arterial contrast filling dynamics on cerebral angiography: An in-vitro pilot study
Source: PLoS One. 2023 Jan 17;18(1):e0280395. doi: 10.1371/journal.pone.0280395 (PMC9844885; doi:10.1371/journal.pone.0280395)
Supplement: Attachment — Submitted filename: Response to Reviewers.docx [file pone.0280395.s001.docx]

**Response to the Reviewers’ comments**

First of all, we would like to thank the reviewers for a thorough reading of the manuscript and helpful comments and suggestions. We have revised our manuscript based on the comments made by the reviewer, and we believe the manuscript is now significantly improved. The followings are the point-by-point responses to the reviewer’s comments.

**Reviewer #1**

This is an excellent pilot study about contrast-filling dynamics in cerebral angiography. I recommend to include in your title: Pilot study.

*Response:* Thank you for your helpful comment. According to your advice, we revised the title of the manuscript as follows: “Feasibility of a silicone vascular phantom replicating real arterial contrast filling dynamics on cerebral angiography: an in-vitro pilot study”

How the authors come up with the idea of a sponge to be used as vascular resistance? Are there any other experiments where they used the same material? Can the authors mention in the paper?

*Response:* Thank you for your thoughtful comment. Unfortunately, there was no prior report on that. Since we found significant reservior effect of a small empty container, we thought that packing the container with sponge would enhance the reservior effect.

**Reviewer #2**

Initially, I would like to congratulate the authors for their interest in advancing in this unexplored and still extremely relevant area. Understanding vascular hemodynamics for in vitro experimentation is an arduous and complex task. Thanks again to the authors for the initiative.

*Response:* Thank you again for your comment. we are very pleased to listen to your appraisal.

**Comments:**

**1. Introduction:**

- Page 3, lines 44-46: change the arrangement of information. Start with in vitro studies and then comment on preclinical work.

*Response:* Thank you for your thoughtful comment. According to your statement, we changed the arrangement of two sentences. Also, some corrections were made to soften the text.

**2. Materials and Methods:**

- Page 3, line 67: specify how many patients were angiographically evaluated and subsequently excluded;

*Response:* Thank you for your helpful comment. From March 1 to June 30 in 2018, total 608 patients underwent the DSA in our institution. Among them, 376 patients were performed to diagnose small size of aneurysm. So, we added that 376 patients were reviewed in the manuscript, and finally 30 cases were carefully analyzed.

- Page 4, line 72: specify how the chosen patient was evaluated. Electronic medical record review? Physical examination?;

*Response:* Thank you for your thoughtful comment. Both angiography and electronic medical records of 376 patients were reviewed and confirmed. Among them, patients with aneurysm in the MCA location were mainly concerned, and as a result, 30 cases were extracted.

- Page 4, line 75: make it clear that only one patient was used in the study;

*Response:* Thank you for your excellent comment. According to your advice, we represented that only one patient data had been utilized in our study exactly.

- Page 4, line 77: emphasize to the reader that the description of 3D printing will be further detailed later in the Materials and Methods section;

*Response:* Thank you for great comment. According to your comment, we added a sentence as follows: “The detailed fabrication description using 3D printing is presented in the Materials and Methods section.”

- Page 5, line 98: when it comes to imaging terms, use descriptors such as "axial, sagittal and coronal planes". In the specific case, coronal plane;

*Response:* Thank you for helpful comment. According to your comment, we added a phrase (“A coronal plane of”) in front of “cerebral DSA images”.

- Page 5, line 116: what type or mixture was obtained for the liquid silicone used? Is this product commercially available or was it manufactured by the authors? If commercially available, add brand and specifications. If manufactured, provide details of the mixture and components;

*Response:* Thank you for thoughtful comment. The liquid silicone material is commercially available (Psycho paint; smooth-on, USA). This product was manufactured by authors and Byung Joon Lee (technical help to develop the cerebral arterial silicone phantom) using this commercial liquid silicone material. As a result, we would like to express my gratitude to him in acknowledgement section.

- Page 7, line 147: Has the 40:60 glycerol/water mixture been validated by previous studies? Clarify why this specific dilution was used;

*Response:* Yes, the ratio of the mixture was represented in the article [11]. In this paper, the following sentence is included: ‘The working fluid was composed of a 40:60 glycerol/water mixture (by weight). The density and dynamic viscosity of the working fluid were 1097 kg·m^−3^ and 3.22 × 10−3 kg·m^−1^·s^−1^, respectively.’ As a result, the dynamic viscosity of the blood-simulating fluid produced for the experiment was 3.22, similar to the patient's dynamic viscosity (3.00~3.22). Therefore, we utilized the same dilution to obtain the same viscosity. As a result, we added two sentences to help the readers to understand the reason clearly.

- Page 8, line 172: Has FWHM been used in other studies? Clarify for the reader how the authors arrived at this parameter and the importance of its use.

*Response:* The FWHM used in this study are very representative value for quantifying contrast concentration curve using contrast medium. It is useful to evaluate the similarity between two peaks of the contrast concentration curves, but it is also important to measure similarity in duration until the next wash-out after reaching that maximum. As a result, we added one sentence to help the readers to understand the reason clearly as follows: “Because it is important to measure similarity in the duration of the contrast concentration until the next wash-out after reaching that maximum, the FWHM was utilized for this purpose.”

3. Results and discussion:

- For better understanding and standardization, consider dividing the sections into Results / Discussion;

*Response:* Thank you for your advice. We divided the sections into Results / Discussion.

- Virtually all data were statistically different from the control group. In this sense, it should be noted that, despite this statistical result, group C was the one with AUC values closer to A. Draw the reader's attention to the favorable data in group C.

*Response:* Thank you for your excellent comment. According to your advice, we added some facts and interpretations in result and discussion section as follows: Results - “Despite this statistical result, type C was the most similar one with the FWHM and AUC values closer to the control group.”, Discussion – “In the case of the maximum contrast concentration and the time-to-peak, type C was not the most similar to that of the control group, but it was relatively marginal from the other two types (A, B). On the other hand, in the FWHM and the AUC, it could be confirmed that type C showed significantly similar values to the control group, unlike the other two groups (A, B).”

4. Minor technical comments:

- Eliminate the use of informal expressions. Example: abstract "to figure out", "simply by"; line 74 "we figured out"; conclusion "there is room for".

*Response:* Thank you for helpful comment. According to your comment, we changed the informal expressions as follows: 1. “to figure out” >> “to analyze”; 2. “simply by” >> “by”; 3. "there is room for" >> “In the future, further modifications to the mechanical details of the proposed approach for variable cerebral hemodynamic conditions seem to be necessary”. Also, some sentences related to this have also been changed to better fit the context.
