# Supplementary material for: Feasibility of a silicone vascular phantom replicating real arterial contrast filling dynamics on cerebral angiography: An in-vitro pilot study
Source: PLoS One. 2023 Jan 17;18(1):e0280395. doi: 10.1371/journal.pone.0280395 (PMC9844885; doi:10.1371/journal.pone.0280395)
Supplement: Attachment — Submitted filename: Response to Reviewers.docx [file pone.0280395.s002.docx]

**Response to the Reviewers’ comments**

First of all, we would like to thank the reviewers for a thorough reading of the manuscript and helpful comments and suggestions. We have revised our manuscript based on the comments made by the reviewer, and we believe the manuscript is now significantly improved. The followings are the point-by-point responses to the reviewer’s comments.

Reviewer #3:

The response analysis considered time to peak and temporal shape of the response, but did not include pure time delay which is critical to control of breathing studies. For this purpose the response should be analyzed with 3 parameters: pure time delay and 2 time constants. See Lange J Appl Physiol 1966:21:1281-1291.

*Response:* Thank you for your helpful comment. In fact, there was no need to analyze the respiration-related time delay because patients held their breath so that the image did not shake during the digital subtraction image acquisition.

Reviewer #4:

1. The manuscript needs a comprehensive rewrite/ review by a native English speaker.

*Response:* Thank you for your thoughtful comment. The revised manuscript that we had already submitted was conducted by a well-known English proofreading company using native speakers. Nevertheless, the English sentences still seem awkward. Therefore, we do one more English proofreading and submit the final version.

2. In the introduction section the problematic and the importance of phantoms development is not clearly defined, moreover the introduction is poorly referenced.

*Response:* Thank you for great comment. According to your comment, the following contents were placed at the beginning of the introduction to supplement the research background.

“Understanding the concentration change of the contrast medium (CM) in cerebral angiography is vital in determining the relationship between contrast concentration and the timing of cerebral blood circulation. However, research on this subject is complicated, as it is ethically inappropriate to repeatedly inject CM into a patient and expose them to radiation. Ahmed et al. represented the bolus geometry of intra-arterial flow by injecting CM into animals [1]. A major limitation of that study was that the hemodynamic conditions among the experimental animals could not be kept constant. After injecting the CM into the animal, recirculation of the CM throughout the animal body may affect the results of subsequent experiment batches [2]. In addition, ethical considerations limit animal testing [3]. In this context, cerebral arterial silicone phantoms that can maintain stable conditions and control external confounding factors such as a constant heart rate, body temperature, and blood pressure would be useful for preclinical evaluation.”

1. Ahmed, A.S.; Deuerling-Zheng, Y.; Strother, C.M.; Pulfer, K.A.; Zellerhoff, M.; Redel, T.; Royalty, K.; Consigny, D.; Lindstrom, M.J.; Niemann, D.B. Impact of intra-arterial injection parameters on arterial, capillary, and venous time-concentration curves in a canine model. AJNR. American journal of neuroradiology 2009, 30, 1337-1341.

2. Burbank, F.H.; Brody, W.R.; Bradley, B.R. Effect of volume and rate of contrast medium injection on intravenous digital subtraction angiographic contrast medium curves. Journal of the American College of Cardiology 1984, 4, 308-315.

3. Ghasemi, M.; Dehpour, A.R. Ethical considerations in animal studies. Journal of medical ethics and history of medicine 2009, 2, 12.

3. The organization of the section material & methods is quite confusing. For example, the sentence (p4, l85) is not clear as we are already in the material & methods section.

*Response:* Thank you for helpful comment. According to your advice, we revised and added the contents (P4, l77 ~ l92) to better understand the patient selection process of the material and methods section as follows:

**Patient selection**

This study was approved by our institutional review board (No. 2020-1013). We analyzed 376 patients who underwent intraarterial catheter angiography in our center from March 1 to June 30 in 2018. The exclusion criteria were as follows: the presence of intracranial stenosis, arteriovenous fistula, or arteriovenous malformation, which can affect the cerebral circulation time; and any other disease, except for an unruptured aneurysm. After the application of the exclusion criteria, 30 cases remained had the same graph pattern as the studies mentioned above [9,10]. Therefore, one patient data among 30 cases was used as a control to represent the shape of the entire CCTC graphs to compare the CCTC with a cerebral arterial silicone vascular phantom (Fig 1). The silicone vascular phantom was manufactured with three-dimensional (3D) printing and mold techniques using the 3D volume data of the selected representative patient. An additional component was attached to reproduce the wash-out of the actual arterial phase. The detailed fabrication description using 3D printing is presented in the “Cerebral arterial silicone phantoms composition” section.

4. A flow diagram will be interesting to understand the patient selection.

*Response:* Thank you for helpful comment. As you suggested, we created a flow diagram to understand the patient selection clearly (P4, l94).

5. P5, l99, reference number does not follow same model as the others numbers.

*Response:* Thank you for your thoughtful comment. We corrected what was mentioned.

6. P7, l153. Would it be possible to perform analysis with others pulse rates? Beats per minute are physiologically at 60, however relatively to the disease it can increase and it would be interesting to investigate the performance with different pulse rate.

*Response:* Thank you for your thoughtful comment. Of the patient data for creating the silicone phantom, the pulse rate was 60 beats per minute. The purpose of this study was to confirm whether the silicone phantom could show a cerebral circulation time similar to that of an actual patient, so it was necessary to implement the same physiologic parameters when obtaining the iFlow image, and for this reason, there was no need to increase the pulse rate.

However, we think the topic you mentioned is an interesting item that can be considered in further research.

7. In results section, please add 95 % for confidence interval or interquartile range.

*Response:* Thank you for your great comment. According to your advice, we added IQR in results section (P9, l216 – l227).
