# Supplementary material for: Feasibility of a silicone vascular phantom replicating real arterial contrast filling dynamics on cerebral angiography: An in-vitro pilot study
Source: PLoS One. 2023 Jan 17;18(1):e0280395. doi: 10.1371/journal.pone.0280395 (PMC9844885; doi:10.1371/journal.pone.0280395)
Supplement: Attachment — Submitted filename: Response to Reviewers.docx [file pone.0280395.s003.docx]

**Response to the Reviewers’ comments**

First of all, we would like to thank the reviewers for a thorough reading of the manuscript and helpful comments and suggestions. We have revised our manuscript based on the comments made by the reviewer, and we believe the manuscript is now significantly improved. The followings are the point-by-point responses to the reviewer’s comments.

Reviewer #3: Separating pure time delay has not been done yet would enhance this paper. Pure time delay is functionally very important to ventilatory control applications.

*Response:* In fact, we recognize that the value of pure time delay you mentioned are functionally very important for ventilation control applications. So, it seems that pure time delay analysis is essential if it is the chest part, or the area closely related to respiration. However, the region of interest in this study is the cerebral artery in the brain, which is relatively insensitive to respiration. In addition, it is judged that the effect on this is small because the image acquisition proceeded with the patient holding the breath.

Also, this study tried to reproduce the actual flow of the cerebral artery with a silicone model, and in particular, it was reproduced to create a shape that gradually decreases after reaching the time-to-peak similarly to the human head. In other words, the most important consideration is to reproduce the time-to-peak value and the wash-out degree similarly (refer to the figure below - Type C is the most ideal). Therefore, pure time delay analysis was not performed separately, and this content has been presented in the discussion section. At this time, we added two reference papers on pure time delay analysis.
