# Supplementary material for: Feasibility of a silicone vascular phantom replicating real arterial contrast filling dynamics on cerebral angiography: An in-vitro pilot study
Source: PLoS One. 2023 Jan 17;18(1):e0280395. doi: 10.1371/journal.pone.0280395 (PMC9844885; doi:10.1371/journal.pone.0280395)
Supplement: Attachment — Submitted filename: Response to Reviewers.docx [file pone.0280395.s004.docx]

**Response to the Reviewers’ comments**

First of all, we would like to thank the reviewers for a thorough reading of the manuscript and helpful comments and suggestions. We have revised our manuscript based on the comments made by the reviewer, and we believe the manuscript is now significantly improved. The followings are the point-by-point responses to the reviewer’s comments.

Reviewer #3: Separating pure time delay has not been done yet would enhance this paper. Pure time delay is functionally very important to ventilatory control applications.

*Response:* Thank you for your great comment, we added the pure time delay values of the control and experimental groups. In addition, two reference papers on pure time delay have been added to the manuscript. As a result, we revealed the pure time delay of type C showed the most constant and stable pattern, so it was possible to reproduce the blood flow of the cerebral artery most similarly compared to other types.
