# Supplementary material for: Feasibility of a silicone vascular phantom replicating real arterial contrast filling dynamics on cerebral angiography: An in-vitro pilot study
Source: PLoS One. 2023 Jan 17;18(1):e0280395. doi: 10.1371/journal.pone.0280395 (PMC9844885; doi:10.1371/journal.pone.0280395)
Supplement: Attachment — Submitted filename: Response to Reviewers.docx [file pone.0280395.s005.docx]

**Response to the Reviewers’ comments**

First of all, we would like to thank the reviewers for a thorough reading of the manuscript and helpful comments and suggestions. We have revised our manuscript based on the comments made by the reviewer, and we believe the manuscript is now significantly improved. The followings are the point-by-point responses to the reviewer’s comments.

Reviewer #3: On page 15 The sentence beginning with: " However, we did not analyze the time delay..." is misleading and should be changed. The time delay as shown by others is not due to respiration but blood flow. The time delay as now measured was about half the time to peak for group C- 2 sec compared to 4.

*Response:* Thank you for your great comment, we deleted the sentence beginning with: "However, we did not analyze the time delay...". In fact, we should have deleted the misleading sentence in the previous revision manuscript, but accidentally failed to make the change. Thanks again for letting us know. In addition, we tried to increase the reliability of the results by adding a sentence showing the difference between the time to peak and the pure time delay by the group in the result part (i.e., 2.27 s; the control group, 1.67 s; type A, 1.61 s; type B, and 1.94 s; type C).
